# Testing the Anticancer Effect of Matcha Using Zebrafish as an Animal Model

**DOI:** 10.3390/nu15102369

**Published:** 2023-05-18

**Authors:** Sara Sokary, Zain Zakaria, Hiba Bawadi, Maha Al-Asmakh

**Affiliations:** 1 Department of Human Nutrition, College of Health Science, QU-Health, Qatar University, Doha P.O. Box 2713, Qatar; sm1509137@qu.edu.qa (S.S.);; 2 Medical and Health Sciences Office, QU-Health, Qatar University, Doha P.O. Box 2713, Qatar; zain.zakaria@qu.edu.qa; 3 Department of Biomedical Sciences, College of Health Science, QU-Health, Qatar University, Doha P.O. Box 2713, Qatar; 4 Biomedical Research Center, Qatar University, Doha P.O. Box 2713, Qatar

**Keywords:** anticancer, general toxicity, matcha, zebrafish

## Abstract

Cancer is the second leading cause of death worldwide, and triple-negative breast cancer (TNBC) patients show the poorest prognosis and survival and the highest metastasis prevalence among all breast cancer subtypes. Matcha has recently been associated with multiple health benefits, and in vitro studies showed the potential effect of matcha in inhibiting cancer development and metastasis. We aimed to determine the safe, non-toxic dose of matcha suitable for zebrafish and to investigate the anticancer effect of matcha on the metastasis and growth of human TBNC cells using a zebrafish xenograft model. Wild-type AB zebrafish were used to conduct multiple general toxicity assessments, including developmental, neuromuscular, and cardiovascular toxicities. The safe, non-toxic concentration of matcha was determined to be 50 µg/mL and 100 µg/mL. Afterward, the zebrafish xenograft model was successfully established for MDA-MB-468 and MDA-MB-231 TNBC cells. The tumor size and metastasis of the injected cancer cells were traced through CM-Dil red fluorescent dye. Upon exposure to matcha at the safe doses, MDA-MB-231 and MDA-MB-468 showed a trend toward reduction in tumor size in a dose-dependent manner, indicated by quantified fluorescence. Matcha also visibly suppressed metastasis of cancer cells in the zebrafish body. Our results point to a potential dose-dependent anticancer effect of matcha on TNBC cells; however, more extended observation periods after xenotransplantation are required to confirm the long-term anticancer effect of matcha on tumor growth and metastasis.

## 1. Introduction

After cardiovascular disease, cancer is the second leading cause of death globally [1], and its incidence and mortality rates have increased over the years [2]. Cancer is defined as unrestrained cell proliferation that acquires metastatic properties in response to oncogene activation and/or tumor suppressor gene inactivation [3]. Breast cancer is categorized according to molecular markers, such as being ER positive (ER+), PR positive (PR+), or HER2 protein-positive (HER2+). A total of 20% of breast cancer is HER2+, and about 70% of breast cancer is ER/PR+ [4]. It can either be negative for all three receptors or positive for two or three at once. The former scenario is referred to as triple-negative breast cancer (TNBC) [4]. TNBC patients mostly show the poorest prognosis and survival and the highest metastasis prevalence among all breast cancer subtypes [5,6].

Matcha is the powder form of Japanese green tea, cultured under special conditions and processed in a unique way after its collection [7,8]. This process allows matcha to have a high content of caffeine and amino acids and lower content of catechins compared to other popular types of green tea [9,10]. However, once dissolved in water, matcha produces three times more catechins compared to the loose-leaf form of green tea [11]. Matcha has grabbed the attention of researchers recently due to its multiple potential health benefits, such as enhancing cognitive function and attention [12], improving lipid profile and lowering body inflammation [13], and acting as an anticancer agent [14]. Research regarding matcha’s ability to fight tumors is in its early stages, with only three in vitro studies conducted to date to investigate matcha’s impact on breast cancer cell. The findings from these studies demonstrate that matcha can considerably impact breast cancer cells’ survival, proliferation, antioxidant response, and cell cycle regulation [14,15,16]. Although evidence from in vitro studies indicates a protective effect against triple-negative breast cancer cells, an in vivo animal model is needed to confirm this effect in a more robust and systematic evaluation of the multicellular connections associated with tumor progression.

In vivo models are valuable for gaining insights into the molecular pathways and clarifying the multicellular connections associated with tumor progression [17]. The zebrafish animal model is considered a good candidate for investigating the effect of matcha on cancer. The resemblance of the zebrafish genome by around 70% orthologue genes makes zebrafish a useful model in genetic manipulation studies [18]. Moreover, zebrafish embryos are simple to maintain as they have a short maturation time, fast organ development, and can produce hundreds of embryos per mating [19]. Due to the fact that zebrafish embryos do not have an adaptive immune system that is active and fully functional until about day 28, this enables the xenotransplantation of human cells in them without fear of rejection, creating a good model for studying human cancer progression and the efficacy of anticancer compounds [20]. Moreover, zebrafish are simple to handle, can be kept in small amounts of water, may be placed in multi-well cell culture plates, and offer a noninvasive cancer model to examine the effects of exposure on the advancement of cancer using high resolution microscopy [21,22,23].

The aim of this study is to determine the efficacy of matcha, administered at a safe, non-toxic concentration, as an anticancer treatment through its effect on two zebrafish xenograft models for TNBC cell lines.

## 2. Materials and Methods

### 2.1. Matcha Extract Preparation

Ceremonial-grade organic matcha was purchased from Wilderness Poets, USA, and stored in a refrigerator at 4 °C. The extract was prepared according to previous literature [24,25] by boiling 50 mg of matcha in 50 mL Embryo medium (E3M) at 150 °C on a hot plate for 20 min, with continuous stirring using a magnetic stirrer. A calibrated sensitive scale was used to weigh the matcha powder, and a 10 mL pipette from Eppendorf was used to obtain an accurate volume of E3M. Before placing the flask on the hot plate, it was covered with foil to prevent water loss through vapor. The extract was centrifuged at 2500 rpm for 3 min, and the supernatant was isolated and used as the stock solution with a concentration of 1 mg/mL. The dilutions were prepared under the hood for sterility. For each experiment, the extract was freshly prepared.

### 2.2. Animal Care and Handling

Animal experiments were all conducted under Qatar University’s Institutional Animal Care and Use Committee (QU-IACUC) approval and in accordance with the national and international guidelines for the use of zebrafish in experimental settings [26]. All experiments followed the animal protocol guidelines required by Qatar University and the Policy on Zebrafish Research established by the Department of Research in the Ministry of Public Health, Qatar. The experimental design for this study was approved by the Institutional Biosafety Committee (IBC) at Qatar University, with the approval document (QU-IBC-2022/014).

### 2.3. Zebrafish Maintenance and Breeding

Zebrafish were housed in recirculating stand-alone aquarium racks, where the water was kept at a temperature of ~28 °C, and the room was kept at 26 °C. The room had a 14-h diurnal light schedule (from 7:30 a.m. to 9:30 p.m.) with 10 h of darkness (9:30 p.m. to 7:30 a.m.). Zebrafish were raised and maintained in normal laboratory settings, as outlined by Westerfield [27], to ensure high-quality embryos. Male and female zebrafish were kept apart in a mating basket the night before mating; the next morning, as the lights were turned on, the barrier was removed, and the zebrafish were left to mate in shallow water undisturbed for about 20 min. The eggs were then collected and cleaned in E3M, which was also prepared according to Westerfield [27]. E3M contains NaCl, KCl, MgSO_4_, CaCl_2,_ and methylene blue in water purified using reversed osmosis. The fertilized and alive eggs were placed in a petri dish with E3M. The embryos were divided into two to three duplicates for each group: control (E3M), positive control (20 ug/mL Zinc Oxide), and the different experimental matcha concentrations. The initially chosen concentrations were based on multiple previous optimization experiments designed to identify the range of the non-lethal concentration of matcha. The eggs were then incubated at a temperature of 28 °C. Up to the time of each experiment, the dead embryos were removed daily with minimal removal of E3M. Only wild-type AB zebrafish embryos aged less than 5 days were used for all conducted experiments.

### 2.4. Developmental Toxicity: Survival Rate and Hatching Rate Analyses

The toxic effect of matcha was determined for each matcha treatment group (i.e., different matcha concentrations) using standard assays, including survival rate and hatching rate. Each group’s dead, surviving, and deformed embryos were monitored and reported every 24 h post-fertilization and until the end of the experiments using a standard examining microscope. The survival rate was calculated as the number of live embryos out of the total number of incubated embryos multiplied by 100. After every 24 h, the dead embryos were removed to prevent causing infection or influencing the survival of the live embryos. Additionally, starting at 48 hpf, the hatching rate was assessed as the percentage of hatched embryos out of the overall number of incubated embryos for each group.

### 2.5. Cardiotoxicity Assessment: Live Imaging of Zebrafish

The anatomy of the zebrafish’s heart is composed of one atrium and one ventricle; the major blood vessels they connect to are the Dorsal Aorta (DA) and Posterior Cardinal Vein (PCV) [28,29]. Several hemodynamic parameters were assessed through live imaging and tracking of the red blood cells’ movement in the DA from the trunk of the larvae. These parameters include cardiac output, blood flow, velocity, vessel diameter, and heartbeat. This was conducted at 72 hpf by stabilizing zebrafish larvae from each treated and control group using 3% methylcellulose on a depression slide with concave wells. Larvae were then positioned correctly to be able to visualize the main vessels in the trunk area. After the larvae were stable, high-speed time-lapse videos were recorded utilizing a Zeiss Lumar V12 stereo microscope equipped with a Hamamatsu Orca high-speed camera and HCImage software. Bright-field videos were recorded for 10 s for each embryo at 100 frames per second and 100× magnification. MicroZebraLab blood flow software from Viewpoint (version 3.4.4, Lyon, France) was utilized to analyze the recorded videos and estimate the blood velocity, vessel diameter, and heartbeat by tracking red blood cells. The fractional shear stress levels were estimated using blood velocity measurements according to Benslimane et al. [30]. The formula (τ = (4 µV_mean_)/D) calculates shear stress (τ, dynes/cm^2^), where µ is the blood viscosity (dynes/cm^2^), V is the average blood velocity (µm/s), and D is the vessel diameter (µm). To calculate the cardiac output (nL/min), the formula (F = V·A) was used, where V is the average blood velocity (µm/s), and D is the vessel diameter (µm). Many published articles explained zebrafish’s heart function analysis technique [29,31].

### 2.6. Neuromuscular Toxicity: Behavioral and Locomotion Assay

To investigate the neuromuscular toxicity of matcha on zebrafish embryos, zebrafish larvae at 96 hpf were individually separated in E3M in a 96-well flat-bottomed plate. The embryos were left in an incubator for one hour to acclimate to the new environment. After acclimatization, the plate was placed in the Viewpoint ZebraLab chamber (Noldus Information Technology, Wageningen, The Netherlands) to automatically track the zebrafish embryos’ movement. The chamber was at 28 °C and was illuminated with white light. The assessment started after a 20 min acclimatization period in the dark; then, alternating 10 min of light and dark were scheduled for 60 min. Calculations of the larvae movement were recorded every 5 min until the end of the experiment. Detection and field settings were adjusted to achieve optimal tracking.

### 2.7. Establish a Xenograft Zebrafish Model

#### 2.7.1. Cell Culture

American Type Culture Collection (ATCC) (Rockville, MD, USA) guidelines were followed for cultivating and maintaining MDA-MB-468 and MDA-MB-231 cell lines (ER^−^, PR^−^, HER2^−^). MDA-MB-468 and MDA-MB-231cells were cultured in RPMI-1640 and DMEM (ThermoFisher Scientific, Waltham, MA, USA), respectively. Both media were supplemented with 10% fetal bovine serum (ThermoFisher Scientific), 1% penicillin/streptomycin antibiotics (Sigma-Aldrich, St. Louis, MO, USA), and 1% L-Glutamine (ThermoFisher Scientific). Cells were incubated at 37 °C with 5% CO_2_ and 76% humidity. Growth media was changed every alternate day to provide the optimum growth environment for cells to continue growing exponentially until they were ready for microinjection. Cells were confirmed to be confluent under a standard microscope before being used in xenograft experiments.

#### 2.7.2. Fluorescent Labeling of Breast Cancer Cells Prior to Xenotransplantation

MDA-MB-468 and MDA-MB-231 cells are adherent cells cultured on a sterile glass coverslip placed in a small petri dish and maintained in growth media for 24 h. The staining solution was prepared using CM-Dil fluorescent dye (V22888, Invitrogen, Waltham, MA, USA), which was freshly prepared for each experiment by adding 1µL of the dye labeling solution to 200 µL of growth media. Once the breast cancer cells were confluent (more than one million cells/mL), growth media was drained off the coverslip. Afterward, 100 µL of the staining medium was placed onto the corner and gently shaken to distribute it over the entire coverslip and put back into the incubator. Several trials were conducted to determine the ideal incubation time for the two cell lines, and the optimum fluorescence and staining results were determined to be after 20 min of incubation with the staining solution. After incubation, the staining solution was removed, and the coverslip was washed with warmed growth media and incubated for 10 min thrice. The cells were then checked under an EVOS M5000 fluorescence microscope (ThermoFisher Scientific, Waltham, MA, USA) with an RFP filter applied in a dark room to confirm uniform staining and adequate fluorescence. Cells were then harvested using trypsinization and pelleted using centrifugation at 1500 rpm for 5 min. The supernatant was discarded, and the cells were re-suspended in serum-free growth media. The cell suspension was then ready to be used in the microinjection procedure. Cells were promptly stained and prepared for each injection trial, and once it was ready, it was used within 2 h. The number of cells contained per milliliter was counted using a Countess 3 Automated Cell Counter (ThermoFisher Scientific) by mixing 10 µL of the cell suspension with 10 µL of trypan blue dye, then loading 10 µL of the mixture to a Countess Cell Counting Chamber Slide (ThermoFisher Scientific). The machine generates the number of alive cancer cells and the percentage of RFP fluorescent cells. Experiments were conducted when more than 80% of the cancer cells emitted RFP fluorescence.

#### 2.7.3. Preparation of Zebrafish for Microinjection

Two days before the microinjection, the adult zebrafish were allowed to breed. The following morning, eggs were retrieved, dead embryos were removed, and the remaining embryos were subsequently incubated at 28 °C. At 2 days post fertilization, the chorion was removed by exposing the larvae to Pronase at 1 mg/mL from a stock of 10 mg/mL, then incubating it for 5 min at 28 °C. Finally, the chorions of any yet unhatched embryos were removed with gentle pipetting using a transfer pipette. Afterward, larvae were washed with fresh E3M three times and incubated at 28 °C until required for injection. At the time of injection, the larvae were transferred to the injection mold and anesthetized by exposing them to 0.003% tricaine methane sulfonate for 2 min, prepared from a stock solution of 4 mg/L concentration, 9 µL of stock was added to 30 mL of E3M. Afterward, zebrafish larvae were placed in the dorsal position in a petri dish coated with agarose gel and molded into furrows. As for the needles used for microinjection, Wehmas’ recommendation [22] was followed, and the borosilicate capillaries were pulled with the Sutter Instrument P-20 at the following settings: pull = 20; velocity = 50; time = 200; air pressure = 200; and heat = ramp +21 °C.

#### 2.7.4. Xenotransplantation of RFP-Labeled Human TNBC Cells in Zebrafish Larvae

MDA-MB-468 and MDA-MB-231 human TNBC cells labeled with red fluorescent dye were xenotransplant into 2-day-old zebrafish larvae. After being redissolved in 200 µL of serum-free DMEM or RPMI-1640, the cancer cell suspension was loaded into the drawn needle fitted with Eppendorf capillary tips. Each needle contained five microliters of the tagged cell suspension. Next, the needle was inserted into the micromanipulator’s orifice. The microneedle was angled at a 45-degree angle. After the drawn needle’s tip was cut using forceps, equal quantities of labeled breast cancer cells were injected into the zebrafish yolk for a total of about *n* = 30 larvae per experimental group. Moreover, 30 larvae were kept un-injected (cancer-free) to serve as the negative control. After the injection, the larvae were allowed to recover for half an hour. Four exposure groups were then created: negative control (cancer-free larvae in E3M), control (injected larvae in E3M), and two matcha concentrations (injected larvae exposed to 50 µg/mL and 100 µg/mL matcha). Larvae were placed in 6-well cell culture plates with 20–30 larvae per group. Although roughly 30 larvae were injected for each treatment group, death, edemas, or having no cancer cells at 1 dpi caused some larvae to be eliminated from further evaluation.

#### 2.7.5. Imaging Breast Cancer Cell Progression in Zebrafish Larvae

At one day post-injection (dpi), the xenotransplant zebrafish larvae were placed on a concave glass slide after being anesthetized in a 0.003% tricaine methane sulfonate solution. To examine the yolk sac, the larvae were placed using a pipette tip in a lateral position to detect the fish with cancer cells and eliminate the ones that did not have cancer cells or were deformed. Imaging was carried out using an EVOS M5000 fluorescence microscope with an RFP filter in a dark room. To assess the proliferation and metastasis of cancer cells as well as the impact of matcha on the number of cancer cells, larvae were photographed comparably at 1 dpi and 2 dpi and compared to the injected control not exposed to matcha. Images were captured at 4× magnification, and a 10× magnification picture was captured for the yolk sac, the injection region.

#### 2.7.6. Quantification of Breast Cancer Cell Fluorescence

To determine the number of cancer cells in the developed xenograft model and demonstrate matcha’s impact on tumor mass size and metastasis, captured images were used to quantify the fluorescent signals emitted by the RFP-labeled TNBC cells. Image J software (National Institutes of Health, Bethesda, MD, USA) was used, and the readings were corrected to eliminate background fluorescence and obtain the corrected total cell fluorescence (CTCF).

### 2.8. Statistical Analysis

Statistical analysis was performed using GraphPad Prism version 9.51 software. Data were analyzed using one way-ANOVA with Dunnet’s multiple comparison test. Statistical significance was considered when the *p*-value was less than 0.05. One asterisk (*) indicates *p* < 0.05, two asterisks (**) indicate *p* < 0.01, three asterisks (***) indicate *p* < 0.001, and four asterisks (****) indicate *p* < 0.0001.

## 3. Results

### 3.1. Developmental Toxicity of Matcha in Zebrafish Larvae

#### 3.1.1. Survival Analysis

**Figure 1:**
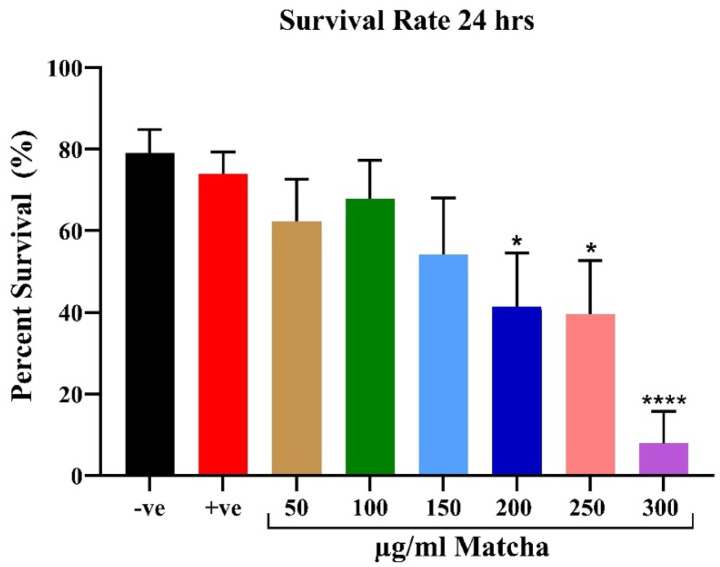
Survival rate at 24 h post-fertilization for various concentrations of matcha. Embryos were visualized and counted using a standard examining microscope. The survival rate was calculated as the number of surviving embryos divided by the total number of embryos used. Data are represented as percent survival (*n* = 30 embryos per group; the experiment was performed in triplicate). Analysis was conducted with one-way ANOVA with Šídák’s multiple comparisons test. * *p* < 0.05, and **** *p* < 0.0001. Abbreviations: −ve, negative control; +ve, positive control.

To determine the safe dose of matcha on zebrafish larvae, multiple concentrations were tested, and the survival rate was calculated every 24 h post-fertilization. Figure 1 shows the survival rate for the various experimental groups after 24 h of fertilization. Matcha concentrations at 200 and 250 µg/mL significantly decreased the survival of the zebrafish embryos (*p* < 0.05). Higher concentrations (300 µg/mL and higher) caused a more profound death rate (*p* < 0.0001). Overall, there was a dose-dependent effect on the survival of the embryos where higher concentrations of matcha decreased survival at 24 h post-fertilization. However, the lower concentration of matcha (≤150 µg/mL) did not significantly affect the survival of the zebrafish larvae. As expected, the positive control (20 µg/mL Zinc Oxide) did not significantly affect the survival rate.

#### 3.1.2. Hatching Rate Analysis

**Figure 2:**
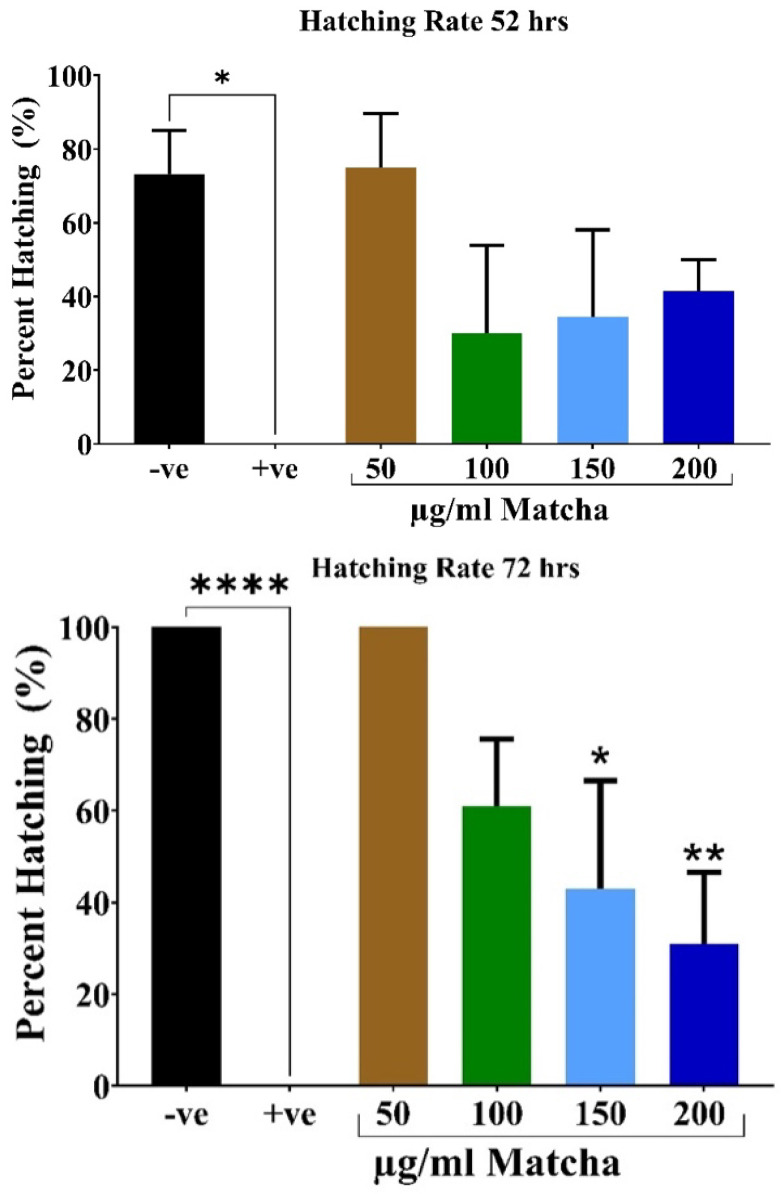
The effect of matcha on the hatching of zebrafish embryos at 52 and 72 h post-fertilization. Embryos were visualized and counted using a standard examining microscope. The hatching rate was calculated as the number of hatched embryos divided by the total number of surviving embryos. Data are represented as percent hatching (%) (*n* = 30 embryos per group; the experiment was performed in triplicate). Analysis was conducted with one-way ANOVA with Šídák’s multiple comparisons test. * *p* < 0.05, ** *p* < 0.01, and **** *p* < 0.0001. Abbreviations: −ve, negative control; +ve, positive control.

The hatching rate represents the number of embryos that hatch from their chorions divided by the total number of surviving embryos. Zebrafish larvae normally start hatching after 48–52 h post-fertilization. Based on the survival results above, the maximum concentration of matcha used to assess the hatching rate was 250 µg/mL. The effect of matcha on zebrafish embryos’ hatching rate, which is usually completed by 72 h post-fertilization, is shown in Figure 2. A dose-dependent decrease in hatching was seen at higher concentrations of matcha, especially at 150 µg/mL concentration and higher. The lower concentrations (100 µg/mL and lower) caused a non-significant decrease in the hatching rate compared to the negative control. The positive control affected the hatching rate by causing 0% hatching of the embryos at all time points, which is the expected effect of ZnO on zebrafish hatching.

### 3.2. Cardiotoxicity Assessment

**Figure 3:**
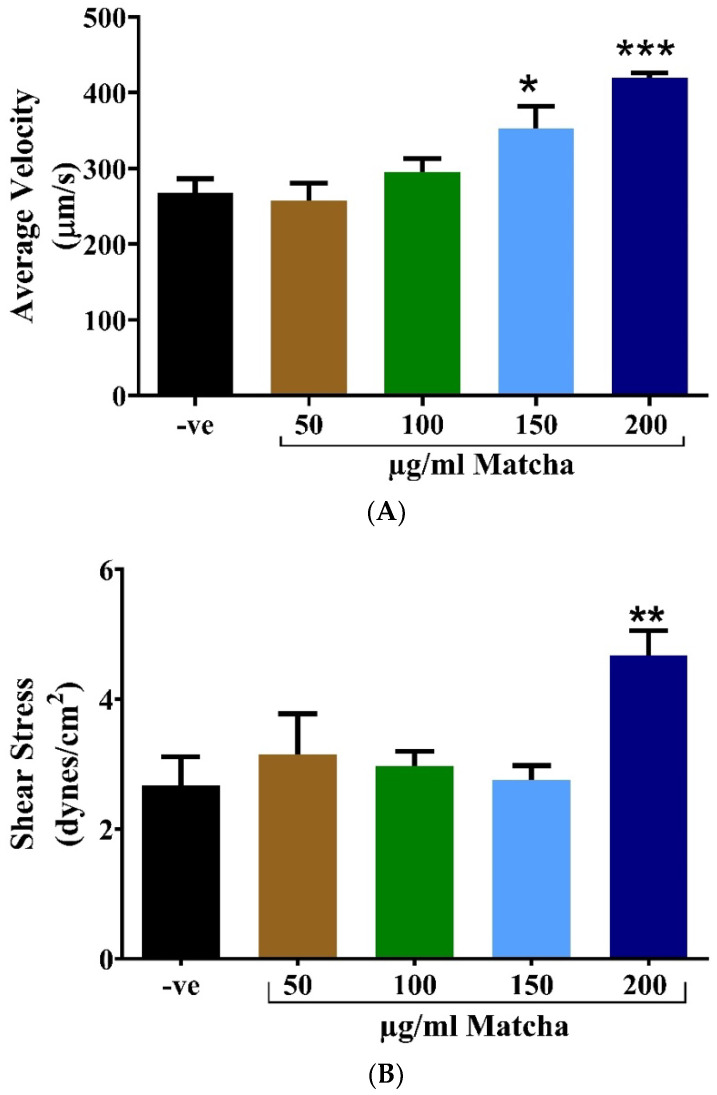

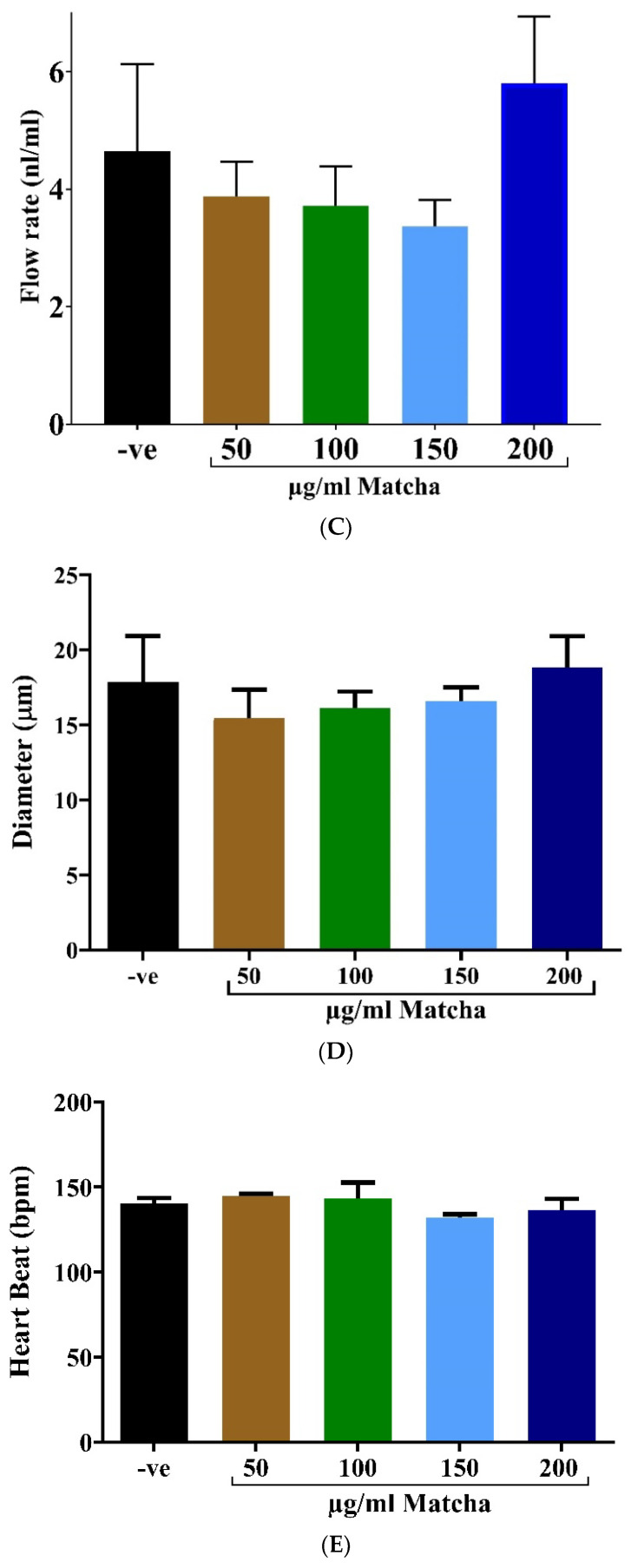
Assessment of the cardiac parameters measured at the dorsal aorta at 72 h post-fertilization. Embryos were stabilized in 3% methylcellulose and visualized using a Zeiss SteREO discovery V8 Microscope equipped with Hamamatsu Orca Flash high-speed camera. A workstation computer and HCImage software were used for video acquisition. A 10-s video at 100× magnification of the trunk region was recorded for each embryo. Videos were analyzed to detect the dorsal aorta blood flow velocity (µm/s) (**A**), shear stress (dynes/cm^2^) (**B**), flow rate (nL/min) (**C**), vessel diameter (µm) (**D**), and arterial pulse (bpm) (**E**). All data are represented as mean ± SEM (*n* = 5 for all groups). Analysis was conducted with one-way-ANOVA with Šídák’s multiple comparisons test. * *p* < 0.05, ** *p* < 0.01, and *** *p* < 0.001. Abbreviations: −ve, negative control; BMP, beats per minute.

Following hatching rate analysis, the cardiac function parameters were assessed on one of the zebrafish’s main vessels, the Dorsal Aorta (DA), a main vessel in zebrafish, at 72 h post-fertilization. As shown in Figure 3A, the aorta blood flow velocity was significantly increased by exposure to 150 µg/mL of matcha (*p* < 0.05), and a more substantial increase was seen with exposure to 200 µg/mL of matcha (*p* < 0.001). Additionally, the aorta shear stress was significantly increased by exposure to 200 µg/mL matcha concentration (*p* < 0.01; Figure 3B). However, the cardiac output (Figure 3C) was not significantly affected by exposure to matcha, although 200 µg/mL of matcha caused a trend toward increased cardiac output compared to the other concentrations, but without statistical significance (*p* > 0.05). No significant difference was detected in the vessel diameter analysis (Figure 3D) and the heart pulse (Figure 3E), showing no specific trend across all matcha concentrations.

According to the results of the cardiotoxicity analysis, a matcha concentration of 200 µg/mL was not considered for further analysis due to its detrimental effect on blood flow velocity and shear stress. Therefore, the concentrations 50, 100, and 150 µg/mL were used further to assess the neuromuscular toxicity of matcha on zebrafish larvae.

### 3.3. Neuromuscular Toxicity: Behavioral and Locomotion Assay

**Figure 4:**
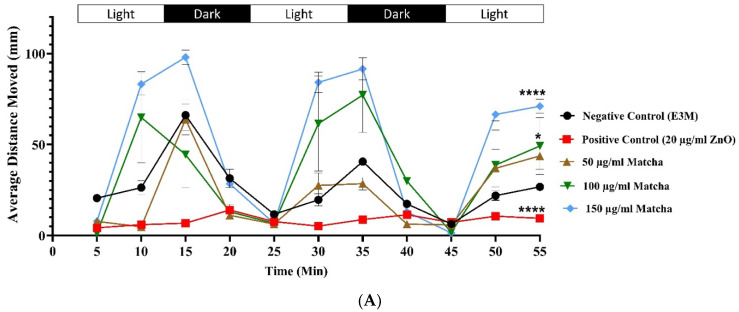

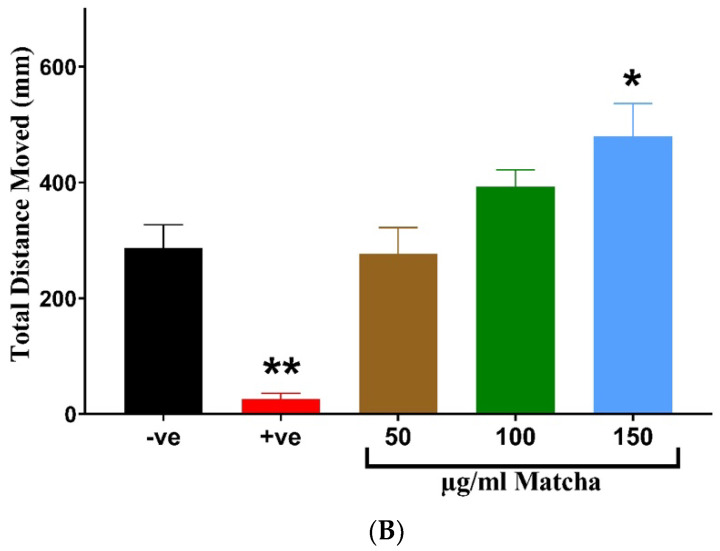
Behavioral and locomotion assay of the experimental groups at 96 h post-fertilization. Single embryos were placed in the Viewpoint ZebraLab chamber and exposed to alternating 10 min light/dark cycles for 60 min. The movement of the embryos was tracked to measure the distance moved by each larva in millimeters. (**A**) The average distance moved by all embryos in each experimental group was measured every 5 min of the assay. (**B**) The total distance moved was measured for all embryos in the exposure group by the end of the assay. All data are represented as mean ± SEM. Analysis was conducted with one-way-ANOVA with Šídák’s multiple comparisons test. * *p* < 0.05, ** *p* < 0.01, and **** *p* < 0.0001. Abbreviations: −ve, negative control; +ve, positive control.

A locomotion assay of the treated versus control groups was conducted to investigate the effect of matcha on the neuromuscular system of zebrafish larvae. The locomotion assay was conducted at 96 h post-fertilization, measuring the average and total distance moved by each larva in millimeters. After an initial 20 min acclimatization period, the measurements were set to be calculated every 5 min during 60 min of alternating 10 min light/dark cycles, as shown in Figure 4A; matcha concentrations higher than 50 µg/mL significantly increased the distance moved by the larvae, which are at 100 and 150 µg/mL of matcha. The effect of 100 µg/mL of matcha significantly increased the average distance moved by the larvae (*p*-value < 0.05).

In comparison, 150 µg/mL caused a higher increase in the average moved distance and more substantial statistical significance (*p*-value < 0.0001). Moreover, the positive control significantly reduced the movement of the larvae when compared to the negative control (*p*-value < 0.0001). Overall, the results showed a regular motion pattern where larvae movement increased in light and decreased in the dark. This pattern was seen for both treated and control zebrafish groups.

Figure 4B shows the total distance moved by the larvae during the locomotion assay. Compared to the negative control, the positive control significantly reduced the total motion of the zebrafish larvae (*p* < 0.01). At the same time, 150 µg/mL of matcha significantly increased the total distance moved by the larvae exposed (*p* < 0.05).

The matcha doses of 50 g/mL and 100 g/mL were non-toxic and did not have any significant harmful effects on zebrafish embryos, as determined by the results of all the toxicity assessments. Consequently, these concentrations were used in the xenograft experiments in an attempt to uncover the anticancer properties of matcha.

### 3.4. Establishing a Zebrafish Xenograft Model

**Figure 5:**
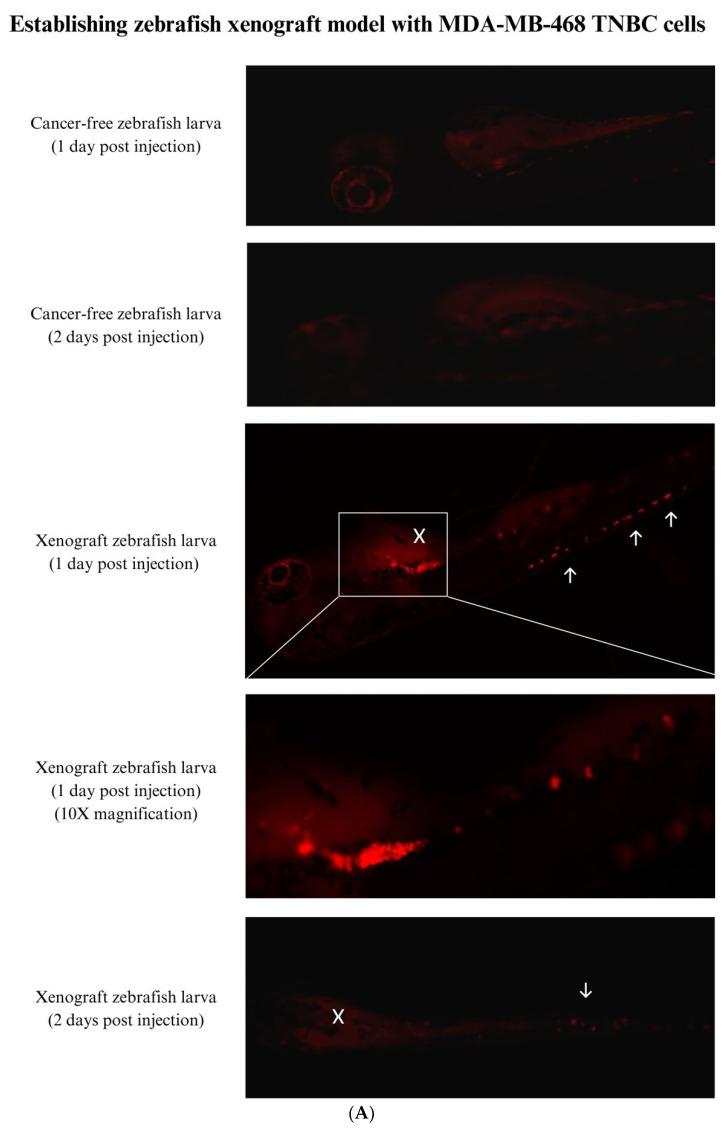

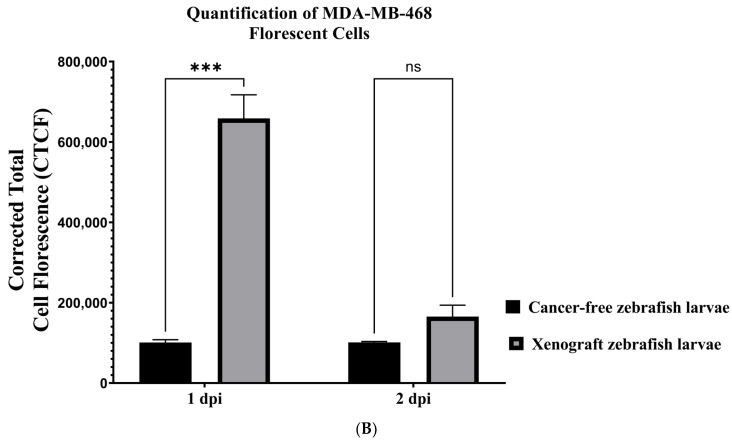
Zebrafish xenograft model injected with MDA-MB-468 cells at 2 days post-fertilization (48 hpf). (**A**) Images of zebrafish embryos xenotransplanted with MDA-MB-468 cell line were taken over a period of 1 and 2 days post-injection; the tumor cell mass was compared to an un-injected negative control embryo. All Images are at 4× magnification unless otherwise stated. White X is the cancer cells’ injection site, white arrows are disseminated cancer cells. (**B**) Quantification of MDA-MB-468 cancer cells inside the xenograft zebrafish compared to the cancer-free control. Abbreviations: dpi, days post-injection. *** *p* < 0.001. ns, not significant.

**Figure 6:**
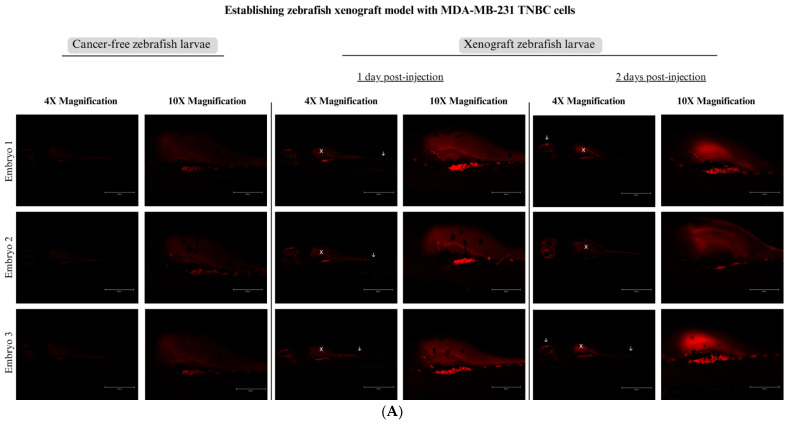

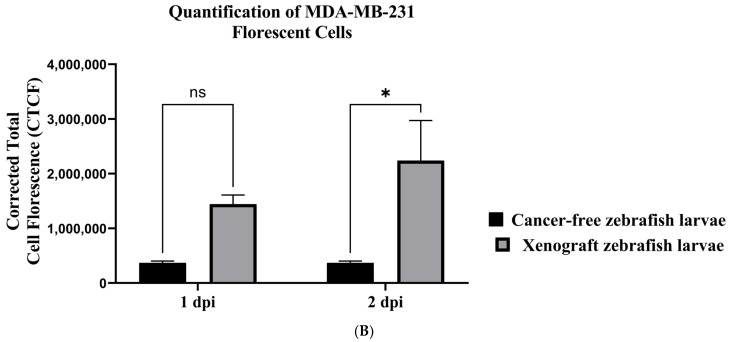
Zebrafish xenograft model injected with MDA-MB-231 cells at 2 days post-fertilization (48 hpf). (**A**) Images of 3 zebrafish embryos xenotransplanted with MDA-MB-231 cell line, taken over a period of 1 and 2 dpi; the tumor cell mass was compared to 3 un-injected negative control embryos. White X is the cancer cells’ injection site, white arrows are disseminated cancer cells. Abbreviations: TNBC; triple-negative breast cancer. (**B**) Quantification of MDA-MB-231 cancer cells inside the xenograft zebrafish compared to the cancer-free control. Abbreviations: dpi, days post-injection. * *p* < 0.05. ns, not significant.

Triple-negative breast cancer cells (MDA-MB-468 and MDA-MB-231) were successfully xenografted into 48 hpf zebrafish larvae. Zebrafish larvae xenotransplanted with MDA-MB-468 (Figure 5A) and MDA-MB-231 (Figure 6A) were imaged at one to two days post-injection (dpi) and compared to cancer-free control larvae. The figures show the differentiation between the embryos’ autofluorescence and the cancer cells’ attachment. At 1 dpi, both cell lines labeled with CM-Dil red fluorescent dye adhered near the injection site, which is the yolk sac. The cancer cells’ injection site (white X) showed the most tumor mass in both cell lines. Both cell lines were able to metastasize to other regions of the zebrafish larvae (white arrows). The cancer cells formed clusters of cells that have the ability to metastasize to different sites, mainly to the tail area. We noticed a decline in MDA-MB-468 cancer cells at 2 dpi, which is a sign that the xenograft zebrafish larvae’s cancer cells failed to survive. However, cells that metastasized to the tail region remained slightly visible. Moreover, MDA-MB-231 cells were able to significantly proliferate and grow in the zebrafish larvae, as seen by increased cancer cells at 2 dpi. The survival rate of the embryos used for injection was constantly above 90%, which is the acceptable survival rate for embryos used in an invasive procedure such as microinjection.

Quantification results revealed successful xenotransplantation of both TNBC cells into 48 hpf zebrafish larvae. MDA-MB-468 showed significantly high fluorescence at 1 day post-injection (*p* < 0.001), revealing successful attachment and homing of cancer cells inside the zebrafish (Figure 5B). However, the fluorescence decreased at two days post-injection, leveling near the cancer-free control and indicating that cancer cells failed to survive in the zebrafish larvae. On the other hand, MDA-MB-231 cancer cells showed high fluorescence at 1 day post-injection, indicating attachment of the cancer cells inside zebrafish larvae (Figure 6B). At two days post-injection, significantly higher fluorescence was observed in xenograft larvae (*p* < 0.05) compared to cancer-free control, reflecting the proliferation and growth of MDA-MB-231 cancer cells inside the xenograft zebrafish larvae.

### 3.5. Xenograft Zebrafish Model Exposed to Matcha

**Figure 7:**
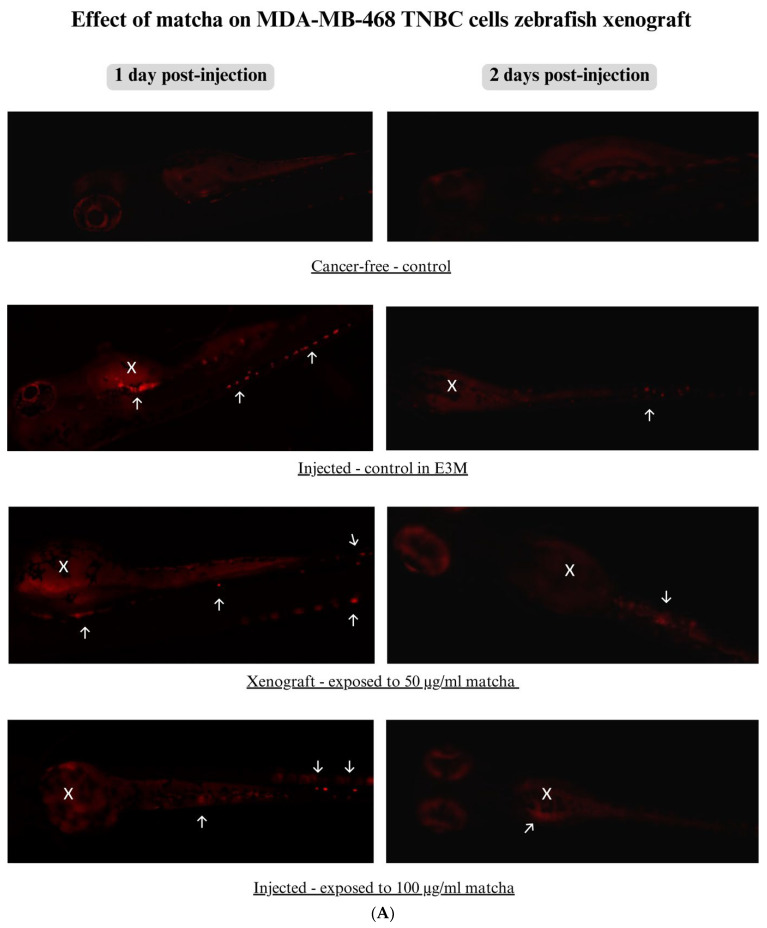

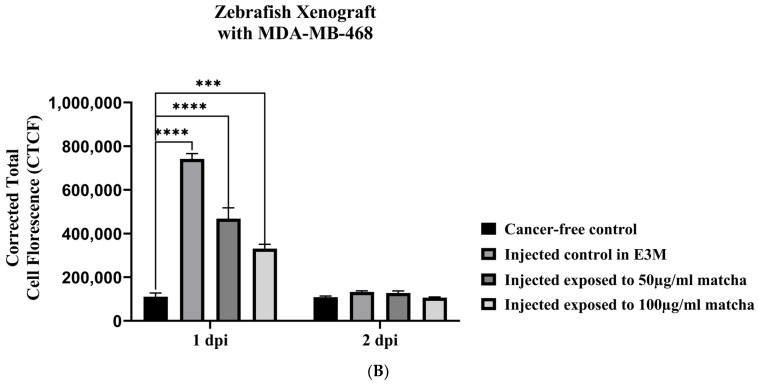
Zebrafish xenograft model injected with MDA-MB-468 cells at 2 days post-fertilization (48 hpf) and exposed to 50 and 100 µg/mL matcha. (**A**) Images of zebrafish embryos xenotransplanted with MDA-MB-468 cell line exposed to 50 and 100 µg/mL of matcha were taken over a period of 1 and 2 days post-injection, and the tumor cell mass and metastasis were compared to an injected control embryo maintained in E3M. White X is the cancer cells’ injection site, white arrows are disseminated cancer cells. (**B**) Quantification of MDA-MB-468 cancer cells inside the xenograft zebrafish compared to the cancer-free control. Abbreviations: dpi, days post-injection; E3M, Embryo medium. **** *p* < 0.0001, and *** *p* < 0.001.

**Figure 8:**
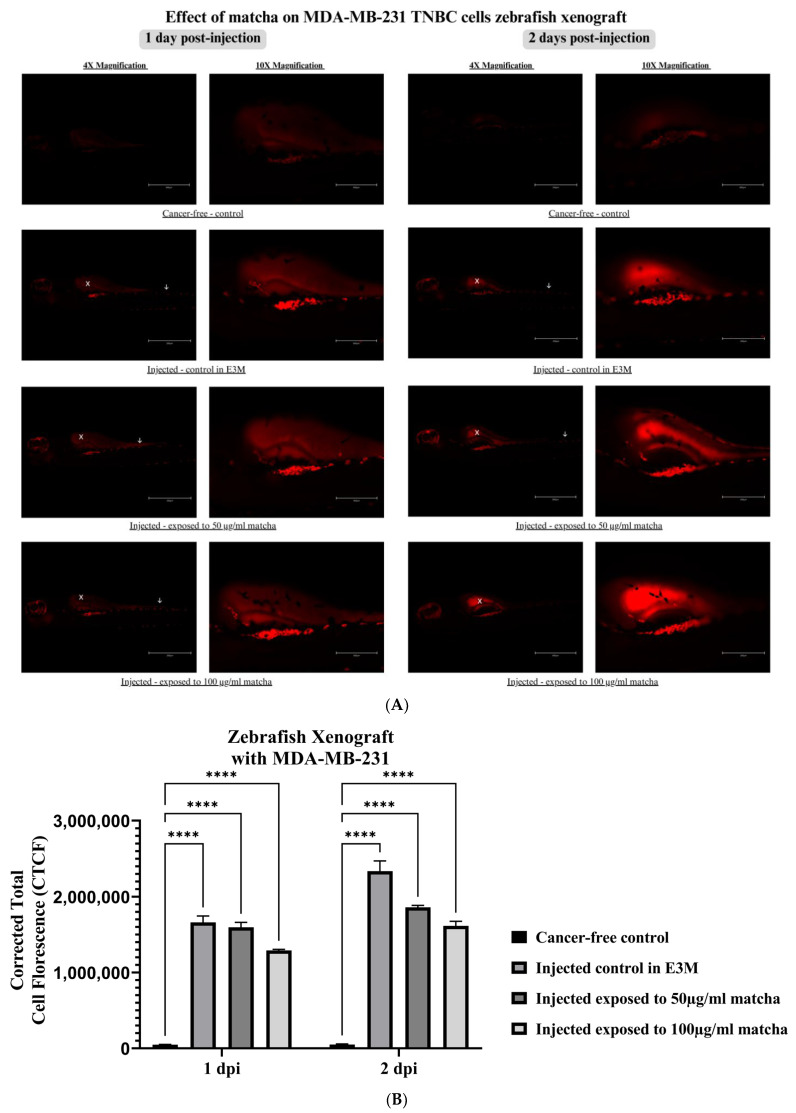
Zebrafish xenograft model injected with MDA-MB-231 cells at 2 days post-fertilization (48 hpf) and exposed to 50 and 100 µg/mL matcha. (**A**) Images of zebrafish embryos xenotransplanted with MDA-MB-231 cell line exposed to 50 and 100 µg/mL of matcha were taken over a period of 1 and 2 dpi, and the tumor cell mass and metastasis were compared to an injected control embryo maintained in E3M. White X is the cancer cells’ injection site, white arrows are disseminated cancer cells. (**B**) Quantification of MDA-MB-231 cancer cells inside the xenograft zebrafish compared to the cancer-free control. Abbreviations: dpi, days post-injection; E3M, Embryo medium. **** *p* < 0.0001.

Xenograft zebrafish larvae were exposed to matcha concentrations at 50 and 100 µg/mL simultaneously with injected xenograft control larvae to assess the anticancer effect of matcha. Figure 7A and 8A show the xenograft larvae exposed to matcha at 1 and 2 days post-injection, compared to injected control maintained in E3M and cancer-free control. The injection site, which is the yolk sac, is indicated by a white X mark, while white arrows mark the fluorescently labeled cancer cells. As seen in Figure 7A, matcha exposure at 50 µg/mL reduced the mass size of the MDA-MB-468 tumor cells at 1 dpi; however, higher suppression was seen at 100 µg/mL matcha. On the other hand, at 1 dpi, migration and metastasis of the cancer cells to the tail area were still visible with exposure to 50 µg/mL but were less visible at 100 µg/mL of matcha. Compared to the injected control, a dose-dependent decrease in tumor mass size was seen with higher matcha concentrations at 1 dpi. An overall decrease in cancer cells was seen at 2 dpi, indicating failure of the cancer cells to survive in the zebrafish larvae.

Figure 8A shows the effect of matcha exposure on the tumor size and metastasis of MDA-MB-231 breast cancer cells. After exposing the xenograft larvae to matcha concentrations at 50 and 100 µg/mL, the tumor size reduced slightly at 1 and 2 days post-injection. The reduction in the tumor size was noted to be dose-dependent, as a higher reduction was seen at 100 µg/mL compared to 50 µg/mL matcha concentration. MDA-MB-231 cells were also able to form clusters of cells and metastasize to the tail region. Matcha exposure at 50 µg/mL did not reduce metastasis of the tumor cells compared to the control; however, 100 µg/mL of matcha was able to reduce the metastasis of the cells to the tail region. The suppression of metastasis was seen more clearly after 2 days of injection, related to longer matcha exposure duration. At 2 dpi, we observed a generally higher background fluorescence in the yolk sac area, which reflects the increased proliferation of tumor cells and growth inside the yolk sac area, also confirmed by quantification data.

Quantification results of fluorescently labeled cancer cells in the xenograft zebrafish control not exposed to matcha showed significantly higher fluorescence compared to the cancer-free control. This xenotransplant cell fluorescence was significantly high in both MDA-MB-468 and MDA-MB-231 cells (*p* < 0.0001). At 1 dpi, MDA-MB-468 xenograft larvae exposed to matcha concentrations at 50 and 100 µg/mL showed a trend toward reduction in the tumor mass size in a dose-dependent manner (Figure 7B). At 2 dpi, all xenograft zebrafish larvae showed a profound reduction in fluorescence, including larvae exposed to matcha, leveling similar to the cancer-free control. On the other hand, MDA-MB-231 matcha-exposed xenograft larvae showed lower fluorescence at 1 dpi compared to xenograft control in a dose-dependent manner (Figure 8B). At 2 dpi, high tumor growth and proliferation were seen in all xenograft larvae; however, matcha-exposed larvae showed a less substantial increase.

## 4. Discussion

Matcha has been gaining popularity recently and is consumed frequently in drinks and confectionary sweets [32,33]. The numerous beneficial health effects of matcha have further increased its popularity. However, the effect of matcha on cancer is an interesting field of study that still requires more investigation. Our study is the first to explore the anticancer effect of matcha using a zebrafish xenograft model. We first identified the optimal concentration of matcha that caused no significant toxic effect on the zebrafish by assessing multiple toxicity assessments. These include the toxic effect on the cardiovascular and neuromuscular systems and the zebrafish larvae’s survival and hatching rates.

The survival of the larvae exposed to matcha was affected by doses higher than 200 µg/mL. Because our study is the first experimental study to assess the effect of matcha on the normal development of zebrafish, our findings are novel. Previous studies examined the effect of different types of teas on the development of zebrafish [24]. However, due to the uniquely higher content of bioactive ingredients in matcha compared to other types of tea [8,34], it is invalid to compare our results. Because matcha is in a powdered form, it allows more release of these bioactive ingredients into the dissolved solvent. Matcha was found to release 3 times more bioactive molecules and polyphenols once dissolved in water compared to other popular teas, including leaf-form green tea [11]. Previous research showed that green tea might have toxic effects on the normal development of zebrafish, but the concentrations they used started from 500 µg/mL of green tea [24], which is ten times higher than the lowest concentration used in our study (50 µg/mL), leading to observed toxic effects.

The hatching of the zebrafish larvae typically takes place 48 to 52 h after fertilization; however, matcha doses higher than 150 µg/mL significantly decreased the percentage of larvae hatching from their chorions even after the 72 h post-fertilization time point. Hatching reflects the normal development of zebrafish embryos, and disruption in the hatching rate is considered a toxic side effect of the treatment. Although bioactive molecules in matcha, such as theanine, caffeine, and catechins, have been associated with multiple health-promoting, disease-preventing, and anticancer effects in the zebrafish model [35,36,37,38,39], high concentrations of these molecules can disrupt the normal development of zebrafish. This is also due to the high sensitivity of zebrafish to any subtle changes in their environment (e.g., PH, conductivity) [40].

Further investigation of the cardiovascular health of the zebrafish was performed by assessing the average blood flow velocity, vessel diameter, shear stress exerted on the vessel, the flow rate, and the heartbeat at the dorsal aorta. Similar to hatching rate results, matcha concentration higher than 150 µg/mL was associated with a significant increase in the blood flow velocity compared to the control. Meanwhile, 200 µg/mL of matcha caused a significant increase in shear stress. Abnormally high blood flow velocity within a major blood vessel, such as the dorsal aorta, suggests vascular abnormalities or defects of the heart muscle [41]. Deviations from the normal values for shear stress (4.04 ± 0.73 dynes/cm^2^), representing the frictional force of flowing blood on the blood vessel and heart walls, indicate cardiac toxicity [42,43]. The two blood flow velocity and shear stress values are related clinically and statistically since the calculations for shear stress include the average blood flow velocity value [30]. Moreover, all the other parameters were not significantly different from the control, and all fell within the normal ranges for dorsal aorta cardiac parameters of a 3-day-old (72 hpf) zebrafish larva, according to a validation study by Benslimane et al. [30]; heart rate: 120–180 bpm, vessel diameter: 15.84 ± 2.5 µm, and flow rate: 5.1 ± 1.1 nL/mL. Matcha concentrations below 150 µg/mL were not associated with cardiotoxic side effects in zebrafish larvae.

To confirm the effect of matcha on the neuromuscular system of zebrafish, a locomotion assessment was conducted by tracking the movement of individual larvae at 96 h post-fertilization while exposing them to alternating light/dark cycles. Zebrafish embryos gain a well-developed neuromuscular system at 96 h, which is when the locomotion assessment was conducted [44]. Our results showed that the movement of the larvae was significantly higher after exposure to 100 and 150 µg/mL of matcha. The higher matcha concentrations showed a dose-dependent increase in the average distance moved by the larvae, which could be due to the higher caffeine concentration the larvae were exposed to, leading to more agitation and alertness in the zebrafish during the light cycles [45]. Caffeine enhances cognitive function, increases alertness and focus, and improves memory [46,47]. Moreover, compared to other types of tea, matcha has a higher caffeine content due to picking only young leaves in the process of creating matcha. Moreover, theanine, a major bioactive compound found in matcha, promotes memory, reduces anxiety, reduces oxidative stress, and protects nervous system function in zebrafish [36]. However, the doses used in this study (33.3 µg/mL) are much higher than the doses used in our experiments (2.3% of matcha’s dry weight, i.e., 2.3 µg and 1.15 µg in 100 and 50 µg/mL concentrations, respectively), which could explain not seeing the anti-anxiety effect of theanine in our matcha-exposed groups. These results were also reflected in the calculated total distance moved by all embryos of the experimental group, where 150 µg/mL matcha concentration showed a statistically significant increase, and the dose-dependent effect was still seen as an increase in the total distance at higher matcha concentrations.

After selecting the safe matcha concentration to test its beneficial anticancer effects, we established different zebrafish xenograft models using MDA-MB-468 and MDA-MB-231 triple-negative breast cancer cells. Both cell lines are a subtype of breast cancer cell line that was derived from the pleural effusion of a patient with metastatic breast cancer. MDA-MB-468 cells present multiple characteristics of breast cancer, such as uncontrolled cell division, invasion, and metastasis [48]. The MDA-MB-231 cell line is also known to be highly aggressive, invasive, and poorly differentiated [49,50]. It is also known to be more metastatic in in vivo models [51] and usually represents a late-stage cancer model [52]. The xenograft zebrafish models were successfully established, indicated by adhering to the xenotransplanted breast cancer cells near the yolk sac and spreading to other areas after 1 day of injection for both cell lines.

MDA-MB-468 breast cancer cells preferred to adhere near the site of injection, which is the yolk sac, and formed clusters once they attached to the home site. The cancer cells were also seen to migrate in clusters to different sites in the zebrafish embryo, such as the tail, which was also seen in previous studies [53,54]. After two days of injection, we observed a profound reduction in cell fluorescence, indicating that MDA-MB-468 cancer cells failed to survive in the zebrafish larvae. Previous studies also tried to establish a xenograft zebrafish model with MDA-MB-468 breast cancer cells. One study was not successful due to the development of severe cardiac edema in >50% of the xenograft larvae [54], while other studies were successful [53,54,55,56]. Studies demonstrated the high adherence and metastasis of MDA-MB-468 cells inside the zebrafish larvae, similar to our results. One study showed that, compared to multiple other types of TNBC cells, MDA-MB-468 was identified as having the highest metastasis potential in the zebrafish larvae [53]. Upon exposure to matcha, tumor cells were seen to decrease in size in a dose-dependent manner, where 100 µg/mL matcha concentration reduced fluorescence signals more than 50 µg/mL. Two days after injection, we observed a reduction in the tumor size at the yolk sac for all xenograft larvae, but a few cancer cells were still fluorescent in the tail area. This was confirmed with fluorescence quantification. More extended observation periods are needed to confirm the long-term effect of matcha exposure on the metastatic activity and tumor growth of MDA-MB-468 breast cancer cells.

MDA-MB-231 cancer cells were also followed up on days 1 and 2 after injection, and the xenograft model was shown to be successful. Tumor cells adhered to the injection site, similar to MDA-MB-468, and could metastasize to other regions in the zebrafish larvae, specifically to the tail area. Previous studies also demonstrated that MDA-MB-231 cells were able to metastasize to the tail area within 24 h post-injection [57]. Other studies also successfully established a zebrafish xenograft model using this cell line [51,57,58,59], and they similarly showed high intravasation as well as extravasation [51,54]. Exposure to matcha reduced the tumor mass size of MDA-MB-231 cancer cells in a dose-dependent manner without statistical significance. However, the metastasis of the tumor cells was more visibly reduced after matcha exposure, where captured images showed that matcha concentration at 100 µg/mL was more able to suppress metastasis of the cells after 1 day of injection compared to 50 µg/mL, and this suppression was more substantial after 2 days of injection. This indicates that MDA-MB-231 cells require higher concentrations and more extended periods of exposure to matcha to show a significant anticancer effect. Further investigation is needed to examine the potential higher tumor growth and metastasis suppression at more extended matcha exposure periods to MDA-MB-231.

When administered at safe levels that cause no adverse side effects in zebrafish embryos and larvae, matcha and its components exhibit multiple health-promoting functions in the zebrafish animal model. One study examined the effect of catechins, which are abundant in matcha, on zebrafish’s oxidative damage and apoptosis in their early development stages. The results confirmed the protective antioxidant effect of catechins by increasing the expression and activity of the antioxidant enzymes in zebrafish [37]. No previous studies examined the effect of a whole matcha extract on the cancer xenograft zebrafish; however, components of matcha were shown to reduce cancer in the zebrafish model [38,55]. The tea pigment theabrownin is a bioactive compound found in all green teas, and it has been reported to have an effect against human cancers in vitro [60,61]. In the zebrafish animal model, a study showed that theabrownin could inhibit tumor growth, increase senescence, and increase apoptosis of hepatocellular carcinoma cells xenotransplanted into zebrafish larvae [38]. Additionally, quercetin is another compound abundant in matcha, where the aqueous extract of matcha contains 1.2 mg/mL, only marginally higher than in traditional green tea (1.1 mg/mL) [39]. Quercetin was proven to be highly effective in suppressing the migration of triple-negative breast cancer cells in zebrafish [55], and its combination with EGCG, the most abundant catechin in matcha, resulted in higher suppression of tumor growth in cell-cultured triple-negative breast cancer cells [16]. This indicates that the holistic effect of matcha may exert more powerful anticancer effects due to the interaction and effects of multiple bioactive compounds. The effect was tested for the first time in our study by observing the effect of matcha on tumor mass size and metastasis in the zebrafish xenograft model. Our results showed a reduction in the tumor size of the MDA-MB-468 cells and suppressed metastasis of the MDA-MB-231 cells xenotransplanted into zebrafish.

Our study has multiple strengths, including using whole matcha and not individually isolated components, which is more relevant to the human consumption of matcha and can help guide recommendations for human cancer prevention. Additionally, we administered matcha for 96 h post-fertilization, which is sufficiently long enough to mimic a human daily consumption lifestyle. Our study is also novel due to being the first research to test the anticancer effect of matcha using an animal model. Additionally, this is the first study to determine the safe, non-toxic dose of matcha suitable for zebrafish to explore its beneficial anticancer effects and successfully determine the safe matcha dose to be 50 and 100 µg/mL.

This study was limited by a few factors, including the complexity of maintenance zebrafish, due to being very sensitive to any subtle changes in temperature, PH, conductivity, and light–dark cycles [40]. Any change in these aspects affects the health of the fertilized embryos and may lead to the absence of any embryo production or the production of low-quality zebrafish embryos after fertilization. Moreover, the fluorescence signal detected from the CM-Dil dye starts to diminish after 30 h of staining [62], which may have affected the observed results at 2 dpi. Finally, in our study, we were able to follow up the xenograft zebrafish for only 2 days post-injection due to the ethical approval obtained.

## 5. Conclusions

Previous literature showed that green tea might be toxic to zebrafish development [24]. Therefore, an emphasis on the toxicity assessment is essential to eliminate potential deviations from the actual results regarding the anticancer effect of matcha tea on the xenograft zebrafish larvae. We were able to test the toxicity of matcha and found that concentrations between 50 and 100 µg/mL were safe and did not cause significant adverse effects in zebrafish. We also successfully established a zebrafish xenograft model using two human triple-negative breast cancer cell lines, MDA-MB-231 and MDA-MB-468. A trend toward reduction in tumor mass size and metastatic abilities was seen after exposure to matcha in a dose-dependent manner. Both cell lines used in this study are highly invasive, metastatic, and resistant to anticancer drugs, and the observed effect of trending toward lower proliferation upon exposure to matcha for only two days is noteworthy and triggers a further investigation. An extended observational period after xenotransplantation of cancer cells is needed to confirm the long-term anticancer effect of matcha and to derive recommendations for human matcha consumption for cancer prevention.

## Data Availability

Data is contained within the article.
